# Biallelic Loss-of-Function Variants in *BICD1* Are Associated with Peripheral Neuropathy and Hearing Loss

**DOI:** 10.3390/ijms24108897

**Published:** 2023-05-17

**Authors:** Yoel Hirsch, Wendy K. Chung, Sergey Novoselov, Louis H. Weimer, Alexander Rossor, Charles A. LeDuc, Amanda J. McPartland, Ernesto Cabrera, Josef Ekstein, Sholem Scher, Rick F. Nelson, Giampietro Schiavo, Lindsay B. Henderson, Kevin T. A. Booth

**Affiliations:** 1 Dor Yeshorim, Committee for Prevention Jewish Genetic Diseases, Brooklyn, NY 11211, USA; 2 Departments of Pediatrics and Medicine, Columbia University Irving Medical Center, New York, NY 10032, USA; 3 Department of Neuromuscular Diseases, UCL Queen Square Institute of Neurology, London WC1N 3BG, UK; 4 Department of Neurology, Columbia University Irving Medical Center, New York, NY 10032, USA; 5 Department of Otolaryngology-Head and Neck Surgery, Indiana University School of Medicine, Indianapolis, IN 46202, USA; 6 UK Dementia Research Institute at UCL, London WC1E 6BT, UK; 7 GeneDx, Gaithersburg, MD 20877, USA; 8 Medical and Molecular Medicine, Indiana University School of Medicine, Indianapolis, IN 46202, USA

**Keywords:** CMT, cytoplasmic dynein, microtubule-based transport, hearing loss, Ashkenazi Jewish

## Abstract

Hearing loss and peripheral neuropathy are two clinical entities that are genetically and phenotypically heterogeneous and sometimes co-occurring. Using exome sequencing and targeted segregation analysis, we investigated the genetic etiology of peripheral neuropathy and hearing loss in a large Ashkenazi Jewish family. Moreover, we assessed the production of the candidate protein via western blotting of lysates from fibroblasts from an affected individual and an unaffected control. Pathogenic variants in known disease genes associated with hearing loss and peripheral neuropathy were excluded. A homozygous frameshift variant in the *BICD1* gene, c.1683dup (p.(Arg562Thrfs*18)), was identified in the proband and segregated with hearing loss and peripheral neuropathy in the family. The *BIDC1* RNA analysis from patient fibroblasts showed a modest reduction in gene transcripts compared to the controls. In contrast, protein could not be detected in fibroblasts from a homozygous c.1683dup individual, whereas BICD1 was detected in an unaffected individual. Our findings indicate that bi-allelic loss-of-function variants in BICD1 are associated with hearing loss and peripheral neuropathy. Definitive evidence that bi-allelic loss-of-function variants in *BICD1* cause peripheral neuropathy and hearing loss will require the identification of other families and individuals with similar variants with the same phenotype.

## 1. Introduction

Numerous genes are fundamental for the development, function, and maintenance of the auditory and peripheral nervous systems. Although these systems differ in many ways, they share many molecular components. Defects in these shared components may affect both systems, such as in some genetic forms of Charcot–Marie–Tooth disease.

Charcot–Marie–Tooth disease (CMT) is a genetically and phenotypically heterogeneous disorder. Clinically, CMT is characterized by chronic motor and sensory polyneuropathy. It can be further refined based on the neuropathy type (demyelinating, axonal, or intermediate), inheritance pattern (dominant, recessive, or X-linked), and the presence or absence of other associated clinical features [1,2,3]. This clinical spectrum results from CMT’s extreme genetic and allelic heterogeneity. To date, more than 100 genes have been associated with CMT [4,5,6,7]. While the exact molecular mechanisms underlying CMT are not fully understood, many of the genes implicated in CMT are essential for intracellular transport [5].

The Bicaudal D (BICD) family of proteins is composed of adaptor proteins that play a role in microtubule-based intracellular cargo transport [8,9]. A common characteristic of this protein family is the presence of multiple coiled-coil domains, which allow for binding to a diverse set of cargos and their coupling with the dynein and dynactin motor complex [9,10]. BICD proteins are critical regulators of the minus end-directed transport of various intracellular organelles [11,12]. Recently, a member of the BICD family, *BICD2*, has been genetically linked to human disease [13,14,15,16,17]. Variants in the *BICD2* gene have been linked to a non-progressive autosomal dominant and progressive autosomal dominant form of spinal muscular atrophy due to heterozygous missense variants (OMIM# 615290) and more severe congenital spinal muscular atrophy due to heterozygous de novo missense variants (OMIM# 618291) [13,14,15,16,17].

Here, we implicate another BICD family member, *BICD1*, which has previously been linked to the modulation of the endosomal sorting of activated neurotrophin receptors in neurons [18] in a human neurological disease. Using exome sequencing, we identified a novel homozygous frameshift variant in the *BICD1* gene, which co-segregated with hearing loss and peripheral neuropathy in a large family of Ashkenazi Jewish descent.

## 2. Results

### 2.1. Clinical Report

**Figure 1:**
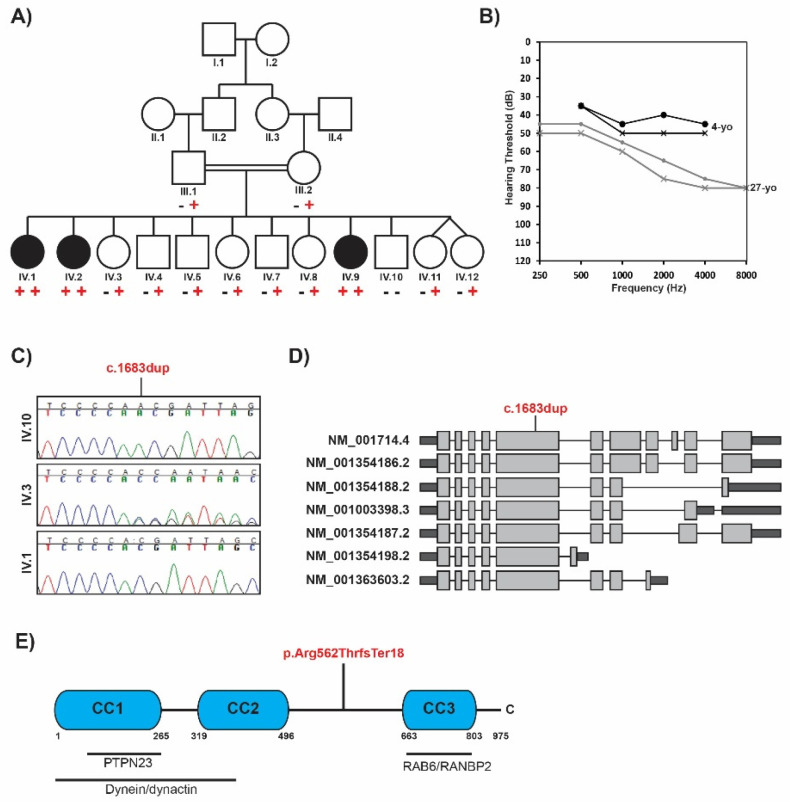
Pedigree, audiograms, schematic representation of the transcripts, and protein of BICD1. (**A**) Family 1 pedigree. Filled symbols denote affected individuals, and double lines indicate consanguinity. A “+” represents the *BICD1* c.1683dup allele segregating with peripheral neuropathy and hearing loss. (**B**) Audiograms of affected individuals VI.1 at 4 years old (black) and 27 years old (gray). Audiograms were obtained using pure tone audiometry with air conduction from frequencies from 250 Hz to 8000 Hz. Circles and “X” denote the right and left ear, respectively. (**C**) Sequence chromatograms showing the wild−type, heterozygous, and homozygous c.1683dup. (**D**) Schematic representation of the transcripts of *BICD1* and the location of the c.1683dup variant in exon 5 of all known RefSeq transcripts. (**E**) Protein schematic illustrating the location of the p.(Arg562Thrfs*18) variant and the interacting partners of BICD1.

Family 1 is a consanguineous family of Ashkenazi Jewish descent. The proband (IV.1), her affected siblings (IV.2 and IV.9), and nine unaffected siblings are offspring of first-cousin parents (Figure 1A).

Both parents are healthy. The proband was born at term after an uneventful pregnancy. Early psychomotor development was reportedly normal. However, her first steps were delayed until she was 3 years old. Hearing loss was first noticed at the age of 3 years and was formally diagnosed at the age of 4. At the time of diagnosis, the severity of sensorineural hearing loss was mild to moderate; however, it has worsened over time and is now in the moderate to severe range (Figure 1B). Interpeak latencies for waves I, III, and V were identified for both ears. The absolute latency for wave I was within normal limits, but waves III and V were delayed, and no inversions of waves were observed. Auditory neuropathy was ruled out using reversed polarity. Distortion production otoacoustic emissions were absent from 1.5 to 12 kHz in the right ear. The left ear could not be measured due to the inability to maintain a seal.

The proband has a history of bilateral hand and foot contractures that progressed slightly over time associated with peripheral neuropathy. However, the patient reports no significant progression in the past decade. She has a history of chronic bilateral leg weakness and numbness. She was clinically diagnosed with Charcot–Marie–Tooth Disease. The physical exam at age 28 years was notable for hand and foot contractures with a full range of motion at the elbows, shoulders, knees, and hips. She had weakness in the distal arms and legs, areflexia at the ankles, reduced sensation to pinprick, and vibration to the mid-shin bilaterally. All the intrinsic muscles in the hands and feet showed atrophy, with pes cavus, hammertoes, and associated unsteady gait. There was electrophysiological evidence of moderately severe, chronic, length-dependent, predominately axonal sensorimotor polyneuropathy. Motor nerve conduction studies of the right median nerve showed normal evoked response amplitudes, prolonged distal motor latencies, and significantly reduced conduction velocity (70% LLN). The right ulnar nerve showed a normal evoked response amplitude, delayed distal motor latency, and reduced conduction velocity at the below-elbow and above-elbow stimulation sites (89% and 79%, respectively). The right fibular nerve showed no recordable evoked response. The right fibular nerve (recording at the tibialis anterior) showed reduced evoked response amplitudes, normal distal onset latency, and mildly reduced conduction velocity. The right second lumbrical–second dorsal interosseous inter-latency comparison showed an abnormally prolonged inter-latency difference affecting the median nerve (1.46 ms). Minimal onset F-wave latencies were prolonged in the right tibial nerve (>114% ULN), right median (>109% ULN), and right ulnar (>118% ULN). Sensory nerve conductions in the right sural, superficial fibular, median, and ulnar nerves showed no recordable evoked responses. The right radial nerve showed a reduced evoked response amplitude and a reduced conduction velocity (89% LLN). Needle electromyograms of select muscles (right tibialis anterior, gastrocnemius, and vastus lateralis) were normal and showed no abnormal spontaneous activity, normal motor unit morphology, and either full recruitment or reduced recruitment on submaximal effort.

Her affected siblings are also products of uneventful full-term pregnancies. Similarly, both had normal psychomotor development, except for delayed walking at 2 and 3 years, respectively. Both report frequent falls in childhood and have unsteady gait and foot drop. Both have mild peripheral neuropathy and have been clinically diagnosed with Charcot–Marie–Tooth Disease. The degree of hearing loss is similar to the proband, and all three siblings use hearing aids. None of the affected individuals have facial dysmorphism or craniofacial issues. All other siblings have normal hearing, displayed normal developmental motor milestones, and reported no clinical abnormalities.

### 2.2. Variant Analysis

Clinical exome sequencing was performed on the proband to identify the underlying genetic cause of hearing loss and peripheral neuropathy in this family. First, we assessed variants in genes associated with hearing loss or peripheral neuropathy. A heterozygous likely pathogenic variant in the *LOXHD1* gene (c.4714C>T; p.(Arg1572Ter)) was identified. Biallelic *LOXHD1* variants are associated with autosomal recessive nonsyndromic hearing loss (DFNB77; OMIM# 613072). However, as no second *LOXHD1* variant was detected, this was thought to represent carrier status only and did not segregate in the other two affected siblings. Given the parental consanguinity, we next prioritized ultra-rare homozygous exonic and splice-site variants for further analysis (Table S1). Limiting the search to non-synonymous variants with less than 1% frequency in the gnomAD and GeneDx databases which were not observed as homozygous in more than three presumably unaffected individuals left 13 variants for consideration. Of these, eleven were missense (three had a damaging Provean score of less than −2.5), one was a non-canonical splice variant at the +4 position (no significant predicted impact on splicing by Alamut), and one was a frameshift variant. Three variants were in known disease genes not associated with hearing loss or peripheral neuropathy, and ten were in genes not previously linked to any human Mendelian disorder. The other two affected siblings underwent exome sequencing and subsequent variant analysis to further narrow down the list of variants. After comparing the final prioritized variant list, the only variant shared in a homozygous state was a novel single base-pair duplication (Chr12:32481072A>A.A. (GRCh37); NM_001714.4:c.1683dup; NP_001705.2:p.(Arg562Thrfs*18)) in the Bicaudal D1 (*BICD1*) gene. Variant confirmation and segregation analysis revealed that the *BICD1* frameshift variant was homozygous in all three affected siblings, heterozygous in both unaffected parents and eight unaffected siblings, and not present in one unaffected sibling (Figure 1A,C). This novel frameshift variant is absent from gnomAD [19]. In the >140,000 individuals in gnomAD, no homozygous loss-of-function variants are reported in *BICD1*.

### 2.3. Carrier Frequency in the Jewish Community

In total, 33,872 Jewish individuals were screened for the c.1683dup variant in the *BICD1* gene. Nine carriers were identified among the 26,086 Ashkenazi Jewish individuals tested, yielding a carrier frequency of 0.017% in this population. However, none of the 4288 Sephardic Jewish individuals or the 3498 individuals of mixed Ashkenazi and Sephardic ancestry were found to be carriers.

### 2.4. RNA and Protein Analysis

**Figure 2:**
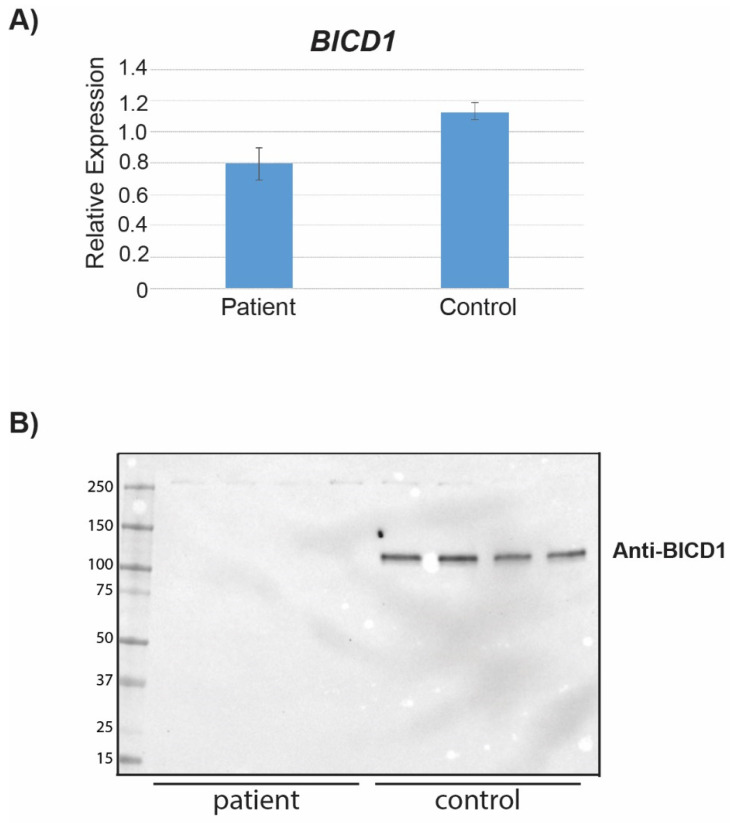
*BICD1* RNA levels and Western blot. RT-qPCR and Western blot of BICD1 from fibroblasts. (**A**) *BICD1* RT-qPCR. Bars indicate standard deviation of ±0.101 and ±0.053 for the patient (IV.1) and control (IV.10), respectively. (**B**) Fibroblasts were collected from an affected individual (IV.1) and an unaffected sibling (IV.10).

The c.1683dup (p.(Arg562Thrfs*18)) variant is located in exon 5 of 10 in the *BICD1* gene and occurs before the third coiled-coiled domain (Figure 1D,E). To explore the impact of this variant on RNA levels, RNA was extracted from fibroblasts derived from one affected individual (IV.1) and an unaffected sibling control (IV.10). The expression of *BICD1* was approximately 30% lower in the patient compared to the control fibroblasts (Figure 2A).

Next, we examined the levels of the BICD1 protein in fibroblasts from an affected individual and an unaffected sibling. BICD1 is a protein of 935 residues with a molecular weight of ~111 kDa. In fibroblasts derived from a heterozygous individual, BICD1 was detected as a single ~111 kDa band (Figure 2B). However, no BICD1 was detected in fibroblasts from the proband, who is homozygous for the frameshift variant (Figure 2B). Ponceau-S staining ensured the equal loading of the lanes (Figure S1). The overexpression of reference His-BICD1 showed the expected ~111 kDa using both the BICD1 (Figure S2A) and His antibody (Figure S2B). In contrast, no protein from the overexpressed mutant (c.1683dup) His-BICD1 construct could be detected with either antibody (Figure S2A,B). Whole protein assessment ensured the equal loading of the lanes (Figure S2C,D).

## 3. Discussion

In this study, we provide genetic evidence that bi-allelic loss-of-function variants in the *BICD1* gene may cause peripheral neuropathy and hearing loss. The *BICD1* gene encodes seven alternatively spliced transcripts. All reported RefSeq transcripts include the first five exons (Figure 1D). The novel c.1683dup variant is located in the large 1095 nucleotide exon five (Figure 1D), resulting in a premature stop in the middle of exon five. The variant was homozygous in all affected individuals and was either heterozygous or absent in the nine unaffected siblings and two unaffected parents.

Based on its location, we expected this variant to cause nonsense-mediated decay [20,21]; however, there was only a 30% decrease in *BICD1* RNA levels (Figure 2A) in fibroblasts derived from a homozygous individual. This is in sharp contrast to protein levels, in which no protein could be detected in fibroblasts from an individual homozygous for the frameshift variant, yet protein was detected in a control individual (Figure 2B and Figure S1). To ensure the frameshift did not impede the BICD1 antibody binding (immunogen residues 538–621), we overexpressed N-terminally HIS-tagged wild-type or mutant BIDC1 constructs in COS7 cells. Neither the BICD1 antibody nor a HIS-tag antibody could detect a BICD1 mutant protein. The patient-derived results and the outcome of the overexpression experiment suggest that the BICD1 mutant protein is most likely degraded or is present at extremely low levels, which are below the limits of detection of Western blotting.

Evidence to support *BICD1* as the causative mutation for peripheral neuropathy and hearing loss is provided from previously described BICD1 mutant animal models. As an adapter protein, BICD1 mediates the coupling of organelles and other cargoes to the minus-end-directed motor dynein/dynactin motor. In *C. elegans* and zebrafish, BICD1 has been shown to be essential in dendritic branching, neuronal patterning, and vesicle transport [22,23]. In addition, BICD1 is essential for the interaction between the plus end motor complex Fignl1-Kif1bβ and dynein/dynactin motor in zebrafish, where Fignl1-Kif1bβ acts as a dynein speed limiter [22]. Perturbing the formation of the dynein/dynactin-BICD1-Fignl1-Kif1bβ complex results in motor axon pathfinding defects. Similar motor axon pathfinding, locomotor behavior, and vesicular transport impairments have been observed in *Kif1b^−/−^* and *Kif1b^+/−^* mice [24]. The phenotype described in the family here, with the p.(Arg562Thrfs*18) variant, could be due to the impaired ability of binding of the FIGNL1-KIF1B complex to the dynein/dynactin motor.

Bicd1 is highly expressed in mice in the developing peripheral and central nervous systems [18]. Expression data from RNA-sequencing data from mouse cochlea at different developmental time points showed that *Bicd1* is expressed more in the sensory cells of the cochlea than the non-sensory cells at embryonic day 16 (E16). Between E16 and post-natal day 0 (P0), there is a decline in *Bicd1* expression (gEAR) [25]. Around P0, *Bicd1* is expressed more in the non-sensory cells of the cochlear epithelium than the sensory cells. Data from the International Mouse Phenotyping Consortium (IMPC) suggest that the *Bicd1* knockout mouse has auditory and neuromuscular dysfunction. Of the 20 different system phenotypes collected by the IMPC, 2 systems (“auditory/vestibular/ear” and “behavior/neurological or nervous system”) are flagged as having significant phenotypes in *Bicd1* knockout mice. In particular, the click-evoked auditory brain stem (ABR) response revealed a significantly increased threshold across all frequencies (*p* = 3.7 10^−6^). *Bicd1* knockout mice are also reported to have a decrease in startle reflex (*p* = 1.9 10^−5^), grip strength (*p* = 2.5 10^−5^), and prepulse inhibition (*p* = 4.7 10^−5^). More detailed analyses are needed to fully characterize the phenotypes in this mouse line.

Studies of ES-cell-derived motor neurons from a *Bicd1*-depleted mouse demonstrated that BICD1 is necessary for the intracellular trafficking of neurotrophin receptors and is a critical modulator of the amplitude and duration elicited by ligand-bound neurotrophin signaling [18]. Specifically, BICD1 controls the BDNF-dependent sorting and progression of p75^NTR^ and TrkB through the endosomal pathway. In the absence of BICD1, the balance between receptor degradation vs. receptor recycling favors receptor recycling [18]. This balance is critical for neurons, as neurotrophin signaling is a key regulator of specification, differentiation, and survival. Unlike the zebrafish and *C. elegans bicd1* knockout models, motor neurons derived from *Bicd1*-depleted mice are morphologically identical to wild-type motor neurons [18,22,23]. This suggests that in mammalian motor neurons, growth factor signaling is the primary defect associated with the loss of BICD1. Normal motor neuron morphology and pathfinding without BICD1 suggests possible partial redundancy between BICD1 and other proteins, including the three other members of the BICD-protein family, which may partially compensate for the loss of BICD1.

More recently, BICD1 has been shown to interact with PTPN23 [26]. PTPN23 is a member of endosomal sorting complexes required for transport and binds BIDC1 via its first coiled-coil domain [26] (Figure 1E). The loss of PTPN23 resembles the cellular phenotype observed upon BICD1 knockdown, resulting in the increased accumulation of p75^NTR^ and TrkB in swollen vacuole-like compartments following BDNF stimulation [26]. Biallelic mutations in *PTPN23* cause a neurodevelopmental disorder and structural brain anomalies with or without seizures and spasticity (NEDBASS) (OMIM# 618890). However, the affected individuals in this study did not display any structural brain anomalies and had normal cognitive function at the time of evaluation.

While the role of BICD1 in the intracellular trafficking of neurotrophin receptors in motor neurons is well studied, BICD1’s role in auditory function is currently unknown. BICD1 may play a similar role in recycling neurotrophin-containing endosomes in the inner ear sensory neurons as in motor neurons. Indeed, BDNF and NTF3 and their receptors (TrkB and TrkC, respectively) are essential for developing and maintaining the afferent innervation of the inner ear [27]. In mice, perturbing BDNF signaling reduces afferent innervation in the apical part of the cochlea, which later progresses to impact all turns of the cochlea [28,29,30]. These mice are deaf and also exhibit progressive hair cell degeneration. Defects in neurotrophin receptor trafficking likely underlie the BICD1-associated auditory dysfunction.

Although the exact mechanism(s) underlying BICD1-related disease still needs to be elucidated, dynein-mediated transport is most likely a key element. Intracellular transport is consistent with the increasing evidence that many genetic forms of CMT are linked to trafficking defects [5]. An in-depth study of the *Bicd1* knockout mouse could elucidate the mechanisms underlying BICD1-related disease in humans and provide further evidence to support the hypothesis that the bi-allelic loss-of-function variants in BICD1 in this family are causative of the observed clinical phenotype.

Heterozygous individuals in this family are clinically unaffected. Additionally, we identified two phenotypically normal carriers in the Jewish population, and loss-of-function variants are present in the gnomAD dataset, suggesting that haploinsufficiency is not a mechanism for *BICD1*-related disease. It is unknown whether dominant-negative or gain-of-function variants cause an autosomal dominant form of BICD1-related disorders, similar to variants reported in *BICD2* [13,15,17]. The genetic, allelic, and phenotypic spectra associated with isolated hearing loss, isolated peripheral neuropathy, and co-occurring hearing loss and peripheral neuropathy are vast. It is possible that other variants in *BICD1* may cause isolated hearing loss or isolated peripheral neuropathy. The screening of *BICD1* should be considered for individuals with co-occurring hearing loss and peripheral neuropathy and in individuals with isolated hearing loss or peripheral neuropathy.

## 4. Methods

### 4.1. Patients

Family 1 was referred to the Dor Yeshorim Committee for Prevention of Jewish Genetic Disease Center in Brooklyn, New York, for genetic testing. After obtaining written informed consent, peripheral blood samples were collected from all affected and unaffected individuals (Figure 1A). Genomic DNA was extracted using standard methods. All affected and unaffected individuals underwent clinical evaluation.

### 4.2. Exome Sequencing and Variant Screening

Exome capture and paired-end sequencing was initially performed on the proband (IV.1) using the clinical exome sequencing pipeline at GeneDx as previously described [31,32,33]. PCR amplification and subsequent Sanger sequencing were used to assess the segregation of the *BICD1* variant in 3 affected and 9 unaffected siblings and both parents. Subsequently, affected individuals IV.2 and IV.9 underwent exome sequencing using the clinical exome sequencing pipeline at GeneDx.

### 4.3. Carrier Frequency in the Jewish Community

The carrier frequency was examined in 33,872 Jewish individuals, with samples genotyped to detect the presence or absence of the BICD1 variant. These anonymous samples were collected through the Dor Yeshorim screening program between May 2022 and February 2023, sourced from various global locations. Dor Yeshorim participants typically encompass all levels of Jewish Orthodoxy. Upon submitting samples for carrier screening, individuals provide written consent for their residual samples to be used anonymously in research to understand single gene disorders within Jewish populations better. Genotyping methods have been previously outlined [34], and individuals from Family 1 served as positive controls.

### 4.4. RNA Analysis

Patient and control skin was biopsied, and fibroblasts were cultured. RNA was extracted from cultured fibroblasts using Qiazol (Qiagen, Hilden, Germany) using the RNeasy Lipid Tissue Mini Kit (Qiagen). RNA was reverse transcribed into cDNA using the RNA to cDNA EcoDryTM Premix (Random Hexamers) (Takara Bio, Shiga, Japan). cDNA from the control and patient samples were PCR amplified using the following primers: F: 5′-TGAAAGGGCCCGATGATCC-3’; R 5′-TGTTCTTGAGATTAGCTAGCGC-3’. This amplifies a 489 bp amplicon that spans from exon 5 to 6 (NM_001717).

qPCR was conducted on a LightCycler 480II (Roche, Basel, Switzerland) using PrimeTime^®^ Gene Expression Master Mix (IDT, Coralville, IA, USA) with primers and probes designed for BICD1 and ACTB as a control in the same tube. BICD1F 5′-CTCCATCTTCAGCAACCTTCC-3’; BICD1R 5′-CTGGAGGCTGAGTACGACA; BICD1probe 5′-6-FAM/TCAAAGAGG/ZEN/CATTTGGGCAGTCCT-3′-IABkFQ (IDT, Cat number Hs.PT.56a.27748968.g). ACTB: F 5’- CCTTGCACATGCCGGAG-3’, R 5’- ACAGAGCCTCGCCTTTG-3’, Probe: 5′-Cy5-TCATCCATG/TAO/GTGAGCTGGCGG-3′-IAbRQSp (IDT, Cat number Hs.PT.39a.22214847). Standard curves for both genes were generated via the serial dilution of the control sample. All measurements were repeated 5 times.

### 4.5. Patient Fibroblast Cell Culture, Lysis and Western Blotting

A punch skin biopsy was performed, and fibroblasts from the proband (IV.1) and an unaffected sibling (IV.10) were cultured in Dulbecco’s Modified Eagle’s Medium (DMEM) supplemented with 10% fetal bovine serum (FBS) and 1% GlutaMAX. Cells were maintained in a humidified 37 °C incubator with 5% CO_2_ and passaged using 0.25% trypsin when 80% confluency was reached. Subsequently, cells were washed with ice-cold PBS while on ice, and proteins (2–4 mg) were extracted using 0.4% NP-40 lysis buffer (50 mM Tris-HCl, pH 7.5, 150 mM NaCl, 1 mM EDTA, 0.4% NP-40, and 5% glycerol), enhanced with a Halt™ protease and phosphatase inhibitor cocktail (1:100; ThermoFisher Scientific, Waltham, MA, USA). Proteins were separated using 4–15% Mini-PROTEAN^®^TGX Stain-Free™ gels (Bio-Rad, Hercules, CA, USA) and transferred to methanol-activated polyvinylidene fluoride (PVDF, Bio-Rad) membranes, following the manufacturer’s guidelines. Ponceau-S staining confirmed equal loading on the membranes, which were then blocked in 5% fat-free dry milk dissolved in PBST for 1 h at room temperature. Subsequently, the membranes were incubated with PA5-59508 primary antibodies (ThermoFisher Scientific) at a 1:1000 dilution in the blocking solution, overnight at 4 °C. After PBST washes, the membranes were treated with suitable horseradish peroxidase-linked secondary antibodies (1:1000; Dako, Glostrup, Denmark) for 1 h at room temperature. The blots were then exposed to enhanced chemiluminescent substrate (Millipore, Burlington, MA, USA) and developed using ChemiDoc™ Touch (Bio-Rad). ImageLab software (version 5.2.1, build 11, Bio-Rad) was used to prepare the images.

### 4.6. BIDC1 cDNA Construct’s Western Blotting

A *BIDC1* cDNA construct containing the coding sequence of transcript 1 (NM_001714) was purchased from ORIGENE (RG218807). The BICD1 cDNA was subsequently cloned into the open reading frame of the pCMV6-AN-His mammalian expression vector (ORIGENE, #PS100011) using SgfI and MluI. Next, site-directed mutagenesis was used to insert the c.1683dup variant. After sequence confirmation, wild-type or mutant constructs were transfected in triplicate into COS7 cells. After 48 h, the cells were lysed, and protein lysate was collected. A total of 20 μg of whole protein lysate was run for 90 min on 4–20% TGX Stain-Free™ (Bio-Rad) gels and transferred onto methanol-activated low fluorescence–polyvinylidene fluoride (LF-PVDF, Bio-Rad) membranes, according to the manufacturer’s instructions. Whole protein was assessed using a stain-free ChemiDoc™ Touch (Bio-Rad) imaging system. Next, the blots were blocked for 1 h and incubated with either with the BICD1-specific PA5-59508 primary antibody or 6x-His tag monoclonal antibody MA1-21315 (ThermoFisher Scientific), each diluted to 1:1000 in blocking solution overnight on a rotator at 4 °C. Following washes with TBST, membranes were incubated with appropriate horseradish peroxidase-conjugated secondary antibodies (1:1000; ABCAM, Cambridge, UK) for 1 h at room temperature. After a final round of washes, membranes were incubated with enhanced chemiluminescent substrate (Biorad) and developed using ChemiDoc™ Touch (Bio-Rad). Images were prepared in ImageLab (version 5.2.1, build 11, Bio-Rad).

## 5. Conclusions

In summary, for the first time, we show that biallelic loss-of-function variants in *BIDC1* are associated with both hearing loss and peripheral neuropathy. To the best of our knowledge, this is the first report linking *BICD1* to a human disease. The identification of additional families with pathogenic variants in *BICD1* and the detailed analysis of *Bicd1* knockout animal models are needed to fully understand the spectrum of disease and confirm a causal association between disease pathogenesis.

## Data Availability

The data presented in this study are available on request from the corresponding authors. The genomic data are not publicly available due to privacy.

## Supplementary Materials

The following supporting information can be downloaded at: https://www.mdpi.com/article/10.3390/ijms24108897/s1.
