# Supplementary material for: Biallelic Loss-of-Function Variants in BICD1 Are Associated with Peripheral Neuropathy and Hearing Loss
Source: Int J Mol Sci. 2023 May 17;24(10):8897. doi: 10.3390/ijms24108897 (PMC10219021; doi:10.3390/ijms24108897)
Supplement: Supplementary file 1 [file ijms-24-08897-s001.zip › ijms-2339177-supplementary.pdf]

Supplementary Figures

Supplementary Figure 1

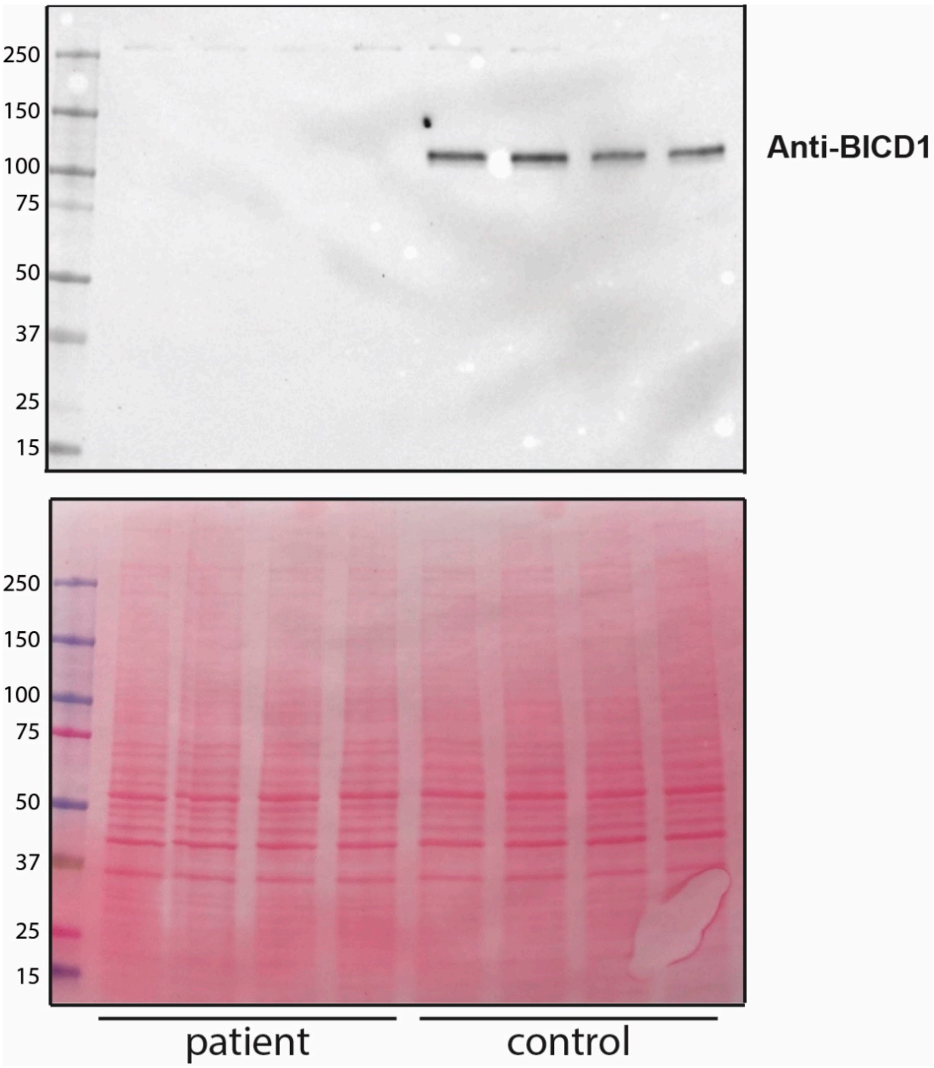

Figure S1. Ponceau-S staining of western blot featured in Figure 2.

Supplementary Figure 2

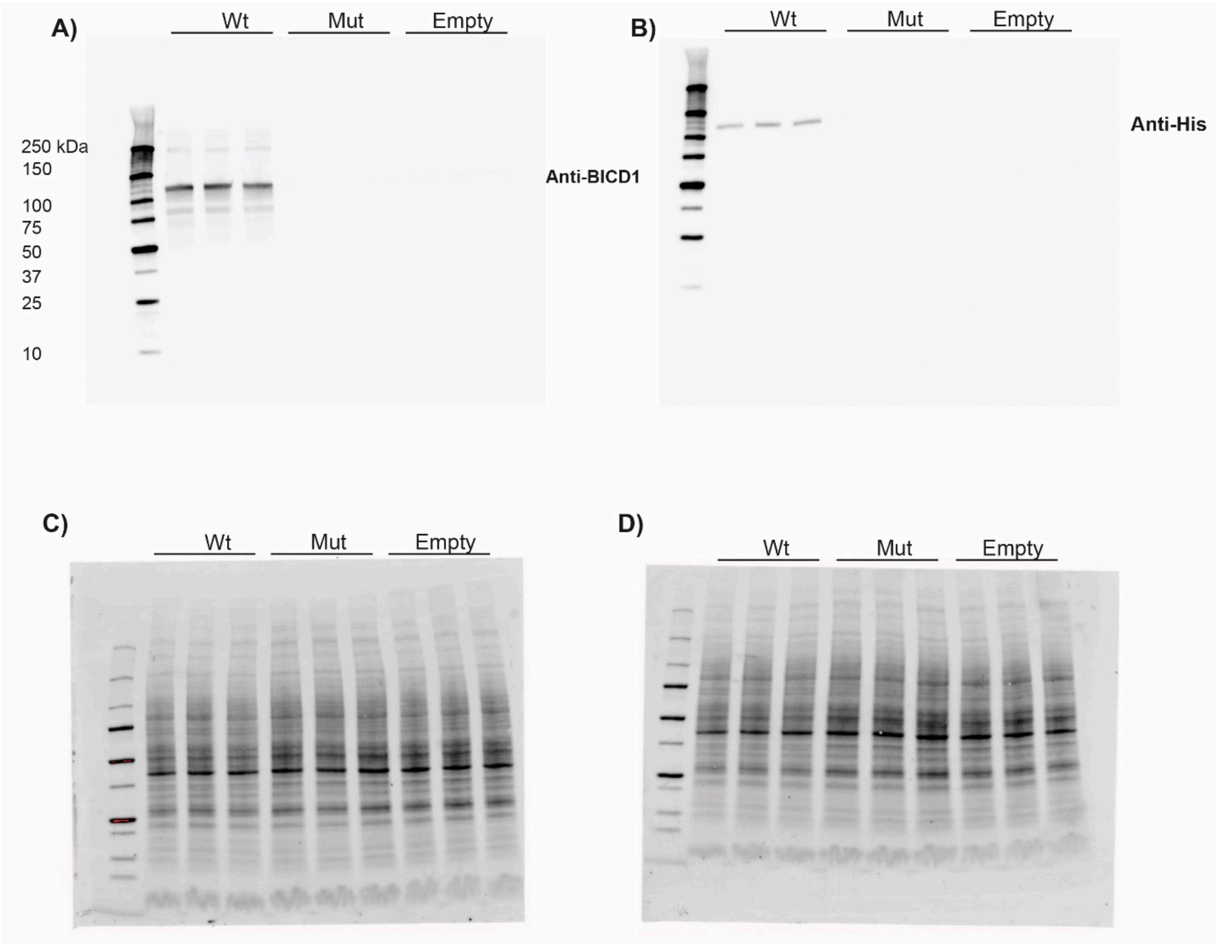

Figure S2. Western blot of His-BICD1 wildtype and mutant from COS7 lysates. Anti-BICD1 stained blot (A) and Anti-His stained (B). Whole protein imaging (C and D) of Anti-BICD1 blot and Anti-His blot, respectively. Note, no detection of the mutant BICD1. Protein maker Precision Plus Protein™ Unstained Protein Standards (Biorad).
